# Discovering fully semantic representations via centroid- and orientation-aware feature learning

**DOI:** 10.1038/s42256-024-00978-5

**Published:** 2025-02-06

**Authors:** Jaehoon Cha, Jinhae Park, Samuel Pinilla, Kyle L. Morris, Christopher S. Allen, Mark I. Wilkinson, Jeyan Thiyagalingam

**Affiliations:** 1 Scientific Computing Department, Science and Technology Facilities Council, Harwell, Didcot, UK; 2 Department of Mathematics, Chungnam National University, Daejeon, South Korea; 3 Electron Microscopy Data Bank, European Molecular Biology Laboratory, European Bioinformatics Institute, Hinxton, UK; 4 Electron Physical Science Imaging Centre, Diamond Light Source, Didcot, UK; 5 Department of Materials, University of Oxford, Oxford, UK; 6 Department of Physics and Astronomy, University of Leicester, Leicester, UK

**Keywords:** Machine learning, Galaxies and clusters, Graphene, Computer science

## Abstract

Learning meaningful representations of images in scientific domains that are robust to variations in centroids and orientations remains an important challenge. Here we introduce centroid- and orientation-aware disentangling autoencoder (CODAE), an encoder–decoder-based neural network that learns meaningful content of objects in a latent space. Specifically, a combination of a translation- and rotation-equivariant encoder, Euler encoding and an image moment loss enables CODAE to extract features invariant to positions and orientations of objects of interest from randomly translated and rotated images. We evaluate this approach on several publicly available scientific datasets, including protein images from life sciences, four-dimensional scanning transmission electron microscopy data from material science and galaxy images from astronomy. The evaluation shows that CODAE learns centroids, orientations and their invariant features and outputs, as well as aligned reconstructions and the exact view reconstructions of the input images with high quality.

## Abstract (web summary)

Cha and colleagues present a translation- and rotation-equivariant autoencoder-based method for robust image recognition, which they demonstrate on diverse tasks from bioinformatics, material science and astronomy.

## Main

The primary challenge in two-dimensional (2D) image analysis in scientific domains is learning meaningful features of objects of interest unaffected by their positions and orientations. In many scientific domains, objects often demonstrate consistent patterns and structural characteristics that can persist even when the objects change in position or orientation. Aligning these objects can enhance the learning of unique, domain-specific features. For instance, 2D projections of proteins in life sciences^1^, four-dimensional scanning transmission electron microscopy data (4D-STEM) data in material science^2^ and galaxy images in astronomy^3^ generally retain their core structural information despite in-plane rotations. However, the arbitrary positions and orientations of objects of interest within an image make it challenging to learn their core structural information without prior knowledge of these attributes. This motivates the need for methods to determine the position (centroid) and orientation of objects in an unsupervised manner, allowing them to be centred in the image and aligned in the same orientation. This enables more efficient learning of object variations independent of centroid and orientation, while simultaneously learning these attributes.

Even though conventional convolutional neural networks have been successfully applied in various image analyses with translation symmetry^4^, many other types of symmetry still occur in 2D images, such as rotation symmetry^3^. Although data augmentation can be considered expedient for addressing these symmetries in neural networks^5^, it can notably increase the training time. Although semantic representation learning is practical via an encoder–decoder architecture, where the features in a lower-dimensional space are enforced to be independent or orthogonal^6,7^, it inherently relies on the ability of the model to understand symmetry^7–9^. Several approaches have been developed in the literature to overcome these issues. For example, a large body of work uses grid sampling in the latent space, explicitly incorporating centroid and orientation information into the decoder^1,10,11^. While grid sampling is applicable to learn centroids and orientations separately, these models require a computationally expensive decoder, limiting their training and inference time capabilities. Moreover, these approaches are not primarily designed to achieve disentanglement in the sense of learning latent dimensions that separately represent distinct features within the data. Instead, they focus mainly on obtaining centroids and orientations to align objects in images.

Here, we propose centroid- and orientation-aware disentangling autoencoder (CODAE), an unsupervised approach that disentangles centroids, orientations and other semantic representations. CODAE includes a translation- and rotation-equivariant encoder to enforce translation and rotation symmetries. Underpinned by relevant theoretical foundations for establishing both translation- and rotation-equivariant and invariant layers, the encoder uses two branches to separately learn the translation- and rotation-equivariant features and their invariant counterparts. Furthermore, we introduce a loss to guide the centroid and orientation feature learning using image moments that address the potential issue of discretization arising from the translation- and rotation-equivariant layers in practice. Moreover, we eliminate the need for a computationally expensive decoder by applying a spatial transformation to the outputs of a decoder, resulting in faster training and inference times compared with the other models. We evaluate CODAE on publicly available datasets from three scientific domains, including life sciences with cryo-electron microscopy (cryo-EM) 2D projections, material science with 4D-STEM data and astronomy with galaxy images. The evaluation shows that CODAE improves semantic representation learning, enabling rapid image interpretation by capturing variations in centroids, orientations and underlying object features. In addition, we compare CODAE against similar approaches that learn centroids and orientations. The quantitative assessment of disentanglement scores on synthetic datasets indicates that CODAE outperforms all models and offers state-of-the-art results. Furthermore, when the dimension of the latent space is too small to transform input data into concise but informative representations, CODAE provides notable additional benefits by reconstructing aligned input images with high quality compared with other models.

## Results

### CODAE architecture

#### Translation- and rotation-equivariant encoder

**Fig. 1:**
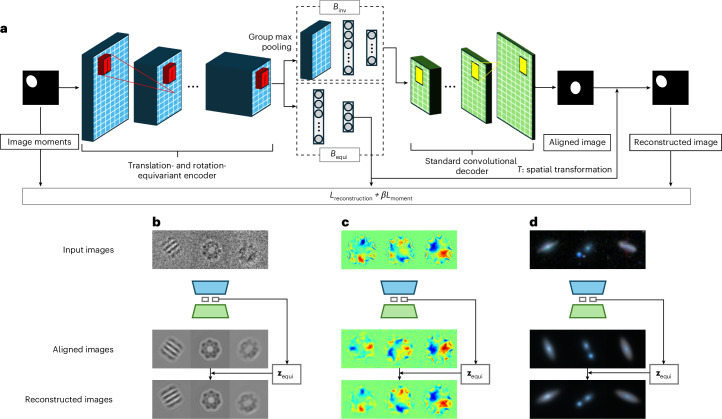
Overview of CODAE architecture and its application to scientific datasets. **a**, An Illustration of CODAE architecture. The model includes two arms and the spatial transformation to learn centroid, orientation and other features effectively. **b**–**d**, The examples of reconstruction of aligned and the same view images of the EMPIAR-10029 (**b**), graphene CBED pattern (**c**) and Galaxy-Zoo datasets (**d**).

CODAE uses a translation- and rotation-equivariant encoder, denoted by *E*_*ϕ*_, that utilizes group-equivariant convolutional layers. These layers apply convolutions with stacks of rotated kernels at equally spaced orientations, enabling the encoder to capture both translational and rotational equivariance in a manner that is analogous to the continuous operation described by Theorem 2 in the Supplementary Information. Following these equivariant layers, the encoder divides into two branches to process either translation- and rotation-equivariant or invariant features. The first branch, denoted by *B*_equi_, is followed by fully connected layers and is specifically designed to learn centroid and orientation factors. The feature maps in *B*_equi_ maintain the same patterns of objects by preserving their centroids and orientations through the equivariant layers, as established by Theorem 2. This allows *B*_equi_ to capture centroid and orientation factors effectively. The second branch, denoted by *B*_inv_, is followed by a group max-pooling layer and fully connected layers. The max-pooling layer enables the learning of features invariant to translation and rotation, as established by Theorem 3 in the Supplementary Information, allowing *B*_inv_ to capture invariant semantic representations. By separately learning centroids and orientation in *B*_equi_ and invariant representations in *B*_inv_, CODAE outputs both aligned reconstructions and the same view images. The proposed network architecture and example reconstructions from three scientific domains are shown in Fig. 1. Please see Methods for model details.

#### Image moment loss

Although the concept of moments was initially introduced in the context of statistics, it has been widely applied in computer vision^12–14^. Moments, which initially describe the distribution of data points, can capture essential features and characteristics of images, including the centre of mass (COM) and orientation of objects. Although image moments capture the centroid and orientation features with a high degree of accuracy, they can still have a degree of subtle inaccuracy. Therefore, we utilize the image moments as guiding principles during the initial stages of learning. Consequently, the loss objective function of CODAE includes the reconstruction loss and image moment loss. To investigate the impact of the image moment loss specifically, we train CODAE with only reconstruction loss. The ablation study in Supplementary Table 4 shows that the image moment loss encourages learning *x* and *y* positions and orientations from *B*_equi_. Gradually decreasing the strength of the image moment loss using a hyperparameter, *β*, during the training, CODAE refines inaccurate centroids and orientations from the image moment. Please see the details of image moments in Supplementary Section A.2.

### Semantic representation learning using CODAE on scientific datasets

To show the capability of learning the semantic representation of CODAE, we test CODAE on publicly available scientific datasets and visualize the latent traversals by varying the values of the target dimension in the latent space while fixing the values in the other dimensions to show individual features that the model has learned.

**Fig. 2:**
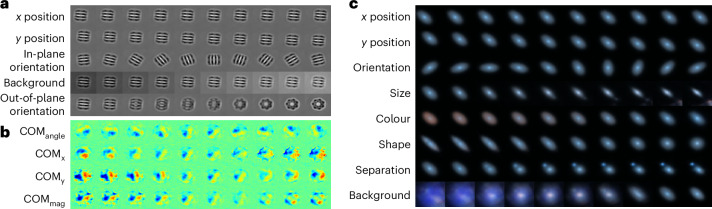
Latent traversals obtained by CODAE across scientific datasets. **a**–**c**, Reconstructions of latent traversals across each latent dimension obtained by CODAE for the EMPIAR-10029 (**a**), graphene CBED pattern (**b**) and Galaxy-Zoo datasets (**c**).

First, we study a simulated cryo-EM dataset (EMPIAR-10029) of GroEL particles, representing the 2D projection images of this specimen imaged using the single-particle analysis approach. The cryo-EM single-particle analysis technique produces low signal-to-noise 2D projection images where the 3D orientation of the molecule or object producing those orientations is unknown. By identifying 2D projections of the molecule taken from similar viewing directions (or object poses) and aligning them through in-plane rotations and translations, one can average these projections. This process amplifies the signal-to-noise ratio to a level suitable for 2D image analysis. Once in-plane rotations and translations are known with sufficient accuracy, out-of-plane object rotations, which produce projections of the molecule in different poses, may be explored in 3D. The averaging of accurately aligned particle images increases the signal-to-noise ratio to reconstruct high-resolution 3D cryo-EM density (Coulomb potential map)^15^. The latent traversal of CODAE shows variations of the particles such as (*x*, *y*)-positions, in-plane orientations, background and out-of-plane orientations (pose) (Fig. 2a). Importantly, the pose representation shows different 2D projections or out-of-plane representations of the 3D molecule. This demonstrates that CODAE could enable image alignment to a reference to perform 3D reconstruction of cryo-EM images or provide priors to other cryo-EM reconstruction refinement algorithms.

To explore the flexibility of CODAE, we next focus on experimental graphene convergent beam electron diffraction (CBED) patterns, where only orientation learning is required, and an alternative to image moments is available. The 4D-STEM technique maps the internal Coulomb potential of a crystal and produces a CBED pattern at each incident electron probe position. The information encoded in the rotational alignment of these patterns includes crystalline orientation and polarization^2,16^. Experimental CBED patterns are typically centred as a preprocessing step, making it unnecessary to learn (*x*, *y*)-positions from the *B*_equi_ branch. In addition, we use the COM to estimate the approximate orientation of CBED patterns rather than image moments, as this is a common approach in CBED pattern analysis, as noted in ref. ^2^. This differs from cryo-EM and galaxy images, where both centroid and orientation variables are learned and compared with approximated centroids and orientations derived from image moments. Following the previous study in ref. ^2^, we chose a 4D latent space and used the Pearson correlation coefficient of the latent features with (*x*, *y*)-components, angle and magnitude of the COM, denoted as COM_*x*_, COM_*y*_, COM_angle_ and COM_mag_ to identify the features learned by CODAE. If the coefficient is less than 0.5, we labelled them as an unnamed feature. By separately learning orientations, *B*_inv_ encodes invariant features that exhibit COM_*x*_, COM_*y*_ and COM_mag_, and the decoder visualizes changes in both *B*_equi_ and *B*_inv_ within real space (Fig. 2b). In addition to the latent traversal, Supplementary Fig. 6 shows the corresponding diffraction patterns, which are proportional to the electric field map that electrons experience as they pass through samples, for each feature, along with the Pearson correlation coefficient. CODAE enables exploring latent features in CBED data beyond the orientation and magnitude typically captured by COM-based methods.

Finally, we explore CODAE on the Galaxy-Zoo dataset. From the Galaxy-Zoo project, many galaxy images became available^17^. As the number of available galaxy images increases, manually annotating these images becomes impractical. Furthermore, the properties of galaxy images remain unchanged by their orientation, which motivates the need for an unsupervised approach to align galaxy images and to learn their semantic representations. In this study, we demonstrate the performance of CODAE using images of 314,000 galaxies from the Galaxy-Zoo dataset^18^. Figure 2c shows that CODAE can visually uncover the dataset’s critical features, such as size, colours, shape, separation and background, by separately learning (*x*, *y*)-positions and orientations. While we provide a visual interpretation of each dimension learned by CODAE in the Galaxy-Zoo dataset, future directions include conducting an in-depth analysis of each latent dimension. This would involve considering galaxy-specific characteristics, such as variations in angular size due to different distances from Earth. Such analysis could further enable CODAE to capture both intrinsic galaxy properties and observational variations, enhancing its utility in galaxy morphology studies.

Our findings demonstrate CODAE’s remarkable ability to learn semantic representations across three diverse scientific datasets, where objects can be freely translated and rotated. This underscores CODAE’s efficiency and highlights its potential to revolutionize understanding and analysing complex scientific data.

### Comparison with other approaches on simulation and scientific datasets

In recent years, encoder–decoder-based architectures have emerged, using spatial transformations to translate and rotate grids in latent space and generating images corresponding to the transformed grids using latent variables. We use two types of evaluation methods: (1) numerical scores on two synthetic datasets, namely, XYRCS (described in detail in Methods) and dSprites datasets^7,19^, to quantify the capability of models to disentangle features, including (*x*, *y*)-positions and orientations, and (2) visualization of aligned images from input images on the two synthetic and three scientific datasets to qualitatively assess alignment consistency. To ensure a fair comparison, we train each model with different random 20 seeds and report the highest total score over these metrics.

#### Numerical scores

**Fig. 3:**
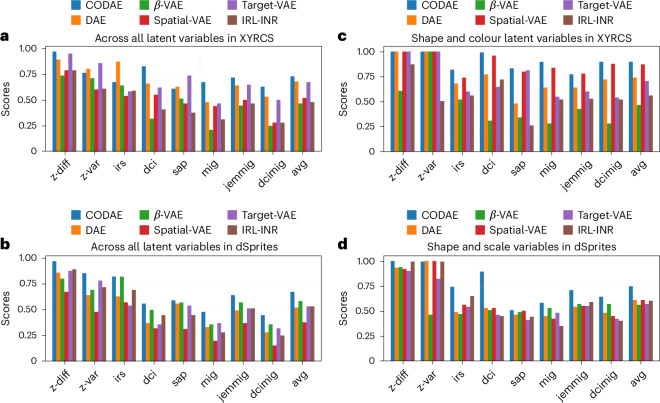
Comparison of supervised disentanglement metrics across models on the XYRCS and dSprites datasets. **a**–**d**, The eight supervised disentanglement metrics of six models on the XYRCS dataset with (*x*, *y*)-positions and orientations (**a**), the dSprites dataset with (*x*, *y*)-positions and orientations (**b**), the XYRCS dataset without (*x*, *y*)-positions and orientations (**c**) and the dSprites dataset without (*x*, *y*)-positions and orientations (**d**).

We report the eight supervised disentanglement metrics on the XYRCS and dSprites datasets (see Methods for datasets and metrics details). Figure 3 shows the evaluation metrics of six encoder–decoder-based neural networks, namely, disentangling autoencoder (DAE)^7^, *β*-variational autoencoder (VAE)^6^, spatial-VAE^1^, target-VAE^10^, invariant representation learning with implicit neural representation (IRL-INR)^11^, and our proposed model, CODAE. DAE and *β*-VAE are not explicitly designed to learn centroids and orientations. Besides, spatial-VAE, target-VAE and IRL-INR are proposed to use spatial transformation in the latent space to learn centroids and orientations (Methods; Supplementary Section A.1 includes details of CODAE and baselines). In both datasets, we set a five-dimensional latent space in DAE and *β*-VAE and a 2D latent space in the other models because the other models explicitly learn (*x*, *y*)-positions and orientations. We report the disentanglement capability of models with and without positions and orientations. Measuring disentanglement scores with positions and orientations offers insights into the capability of fully semantic representation learning. Then, positions and orientations are identified among five features and excluded to measure disentanglement scores. These scores give insights into the capability of learning patterns and structures independent of positions and orientations. When considering the average of all metrics, CODAE substantially outperforms baselines. For the XYRCS dataset, target-VAE performs as well as CODAE with all features, while spatial-VAE exhibits a similar level of performance to CODAE in colour and shape feature learning. For the dSprites dataset, CODAE outperforms all models across all metrics when all features are considered. With only scale and shape features, it outperforms the other models except for the z-var (described in Methods) score. However, the difference between the best performance and the performance of CODAE in the z-var score is marginal and negligible.

#### Visualization of aligned images from input images on two synthetic and three scientific datasets

**Fig. 4:**
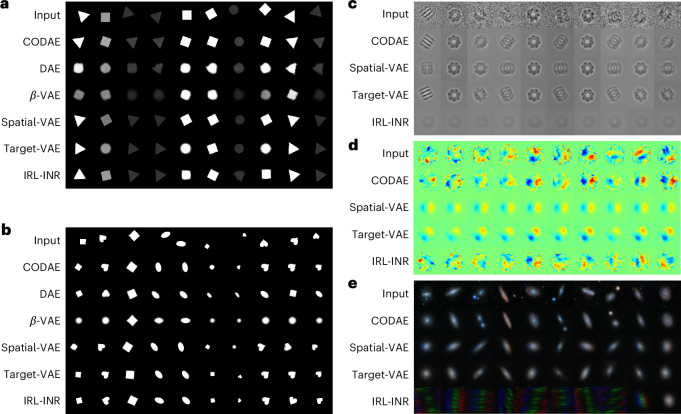
Comparison of aligned and reconstructed images across models on five datasets. **a**–**e**, The visualization of aligned images from input images on the XYRCS (**a**), the dSprites (**b**), the EMPIAR-10029 (**c**), the graphene CBED patterns (**d**) and the Galaxy-Zoo datasets (**e**). The aligned reconstructed images are generated by each model using the centroids and orientations learned from the model.

We use (*x*, *y*)-positions and orientations, which are identified after training, to investigate further the capability of learning centroids and orientations of models to generate aligned reconstruction across images in a dataset. As shown in Fig. 4, DAE and *β*-VAE struggle to align them in the XYRCS and dSprites datasets without alteration. Meanwhile, for the XYRCS dataset, CODAE and spatial-VAE perfectly align all objects. Target-VAE cannot align the squares, and IRL-INR faces difficulties keeping shapes when the object is either a circle or a rectangle. For the dSprites dataset, while CODAE aligns images by preserving their scale and shape, the other models fail to align heart images except for target-VAE. The scientific datasets lack ground-truth factors, making it difficult to manually identify or verify (*x*, *y*)-positions and orientations. For this reason, we report the aligned reconstructions of the EMPIAR-10029, experimental graphene CBED patterns and Galaxy-Zoo datasets for only CODAE, spatial-VAE, target-VAE and IRL-INR models. First, in the EMPIAR-10029 dataset, CODAE, spatial-VAE and target-VAE can reconstruct visible and aligned images compared with IRL-INR as shown in Fig. 4c. Next, Fig. 4d shows that spatial-VAE and target-VAE align images with the same orientation but fail to reconstruct the original graphene CBED patterns. Although IRL-INR outputs different patterns based on the inputs, the aligned patterns are not as sharp as those reconstructed by CODAE. Finally, Fig. 4e shows that the aligned galaxy reconstructions produced by CODAE are visually better than those of other models, especially when two galaxies are present in the image. Although CODAE outperforms the others, it still smooths the galaxy structure. This smoothing results in a loss of complex structures, but it is worth noting that it may be beneficial for approximating symmetric light profiles for galaxies.

Our work revealed that, while most models can reconstruct aligned images in the XYRCS, dSprites and EMPIAR-10029 datasets, the CODAE model stands out as the only one that aligns input images with high quality in the graphene CBED pattern and Galaxy-Zoo datasets. In addition to the aligned reconstruction of each model, latent traversals of all models are included in Supplementary Section A.3.

## Discussion

Identifying better representations is essential for extracting meaningful insights from noisy, complex data across scientific domains. With the development of techniques, analysis of a large volume of 2D images is an increasingly important domain for applying deep learning. More specifically, during a single session in a cryo-EM experiment, a few thousand micrographs can be generated, and each includes several hundred particle projections^15^. In material science, the recent development of high-speed direct electron detectors enables the collection of large 4D-STEM datasets in which 2D diffraction patterns are recorded over several tens of thousands of electron probe positions^20^. In astronomy, observational surveys are generating increasingly detailed galaxy images for studying galaxy formation and evolution. For example, in the near future, the Vera Rubin Observatory will carry out the Legacy Survey of Space and Time, which will provide photometric images of at least 10^10^ galaxies^21^.

To enforce some symmetries in image analysis in different scientific domains, many recent works have proposed to enforce both translation and rotation symmetries by using a spatial transformation in the latent space, which translates and rotates the Cartesian coordinates centred on the image to align with the centre of the object in the image, before passing it, along with other latent variables, to the decoder^1,10,11^. While a spatial transformation in the latent space is straightforward to implement, applying a spatial transformation in the latent space can be computationally expensive, particularly when combined with a feedforward network-based decoder. To address this, CODAE uses a translation- and rotation-equivariant encoder and applies a spatial transformation to the decoder output to translate and rotate aligned reconstructions, rather than manipulating the Cartesian coordinates in latent space, as done in models such as spatial-VAE, target-VAE and IRL-INR^1,10,11^. This enables the use of a conventional 2D convolutional decoder.

The pair of a translation- and rotation-equivariant encoder and a conventional decoder offers exceptional training and inference time. Supplementary Table 7 presents the training and inference time of all the models across three different graphics processing unit architectures, namely, *V*100, *A*100 and *H*100. The results show that the training and inference time of the proposed model are at least 10× and 8× faster than the average performance of other models, respectively. The enhanced encoder design also allows Euler encoding between the translation- and rotation-invariant layer and the conventional decoder as CODAE applies the spatial transformation to the decoder output rather than within the latent spce. This enables CODAE to effectively encode variations independent of centroids and orientations, including shape, scale and brightness attributes.

The ablation study found that both translation- and rotation-equivariant encoder and image moments loss contribute to learning centroids and orientations. When measuring disentanglement scores with (*x*, *y*)-position and orientation features, replacing translation- and rotation-equivariant layers with conventional convolutional layers results in a 10% and 44% decrease in the average score on the XYRCS and dSprites datasets, respectively. In addition, disabling the image moment by setting *β* to zero results in 32% and 25% drops in the average score on the XYRCS and dSprites datasets, respectively. In addition, Supplementary Table 1 shows that the disentanglement scores for centroid and orientation learning using image moments are lower than those achieved with the proposed approach. Future studies will include the impact of the *β* value for image moment loss in learning centroid and orientation features to further explore the advantages of CODAE over image moments. For the ablation study, scores overall metrics are presented in Supplementary Table 4.

Our evaluation shows that CODAE extracts independent features that describe each aspect of objects while accurately reconstructing the input images across different scientific domains. In particular, the Galaxy-Zoo example in Fig. 4e highlights the potential use of CODAE when two objects are present in the images, whereas other models are more reliable when only one object is present. While this Article focused on latent traversals to visualize the learned features, CODAE can potentially help scientists quantitatively analyse their data in the future. This could be applied to determining in-plane orientation in cryo-EM experiments and quantifying the distances between two galaxies in astronomy. In addition, while the current method is limited to translation and rotation, extending it to include depth and scale would broaden its applications to a wider range of scientific domains.

## Conclusion

Learning distinct features, most importantly centroids and orientations, of objects in 2D images is crucially important for a number of image applications, such as aligned image reconstructions and learning pose-invariant features, in various scientific domains. Although the notion of translational and rotational equivariance and invariance in neural network-based models is very important, possessing these properties does not guarantee that such models learn centroids and orientations, especially along with other features.

This Article utilized an approach that learns orthogonal features, including centroids and orientations. By relying on translational and rotational equivariant layers and image moments, we showed that CODAE learns equivariant and invariant features related to translation and rotation. Our evaluation demonstrated that CODAE can offer superior performance in learning centroids and orientations along with other features. More specifically, the superior quality of the outputs for aligned reconstructions and reconstructions of latent traversals across three scientific datasets showed the full potential of the proposed approach. We hope that the proposed model will have real-world utility in addressing several scientific problems.

Although the results are of superior quality, we believe there is room for further research. Specifically, we are interested in assessing the impact of image moments on noisy datasets, which would lead to a better understanding of the potential applications, albeit the fact that this is a separate study.

## Methods

### Datasets

We used the XYRCS and dSprites datasets that contain reliable ground-truth labels^7,19^. The XYRCS dataset is a simple yet effective synthetic dataset containing three shapes (a circle, a triangle and a rectangle) with varying *x* and *y* positions, orientations and colour information (specifically, the brightness). The dSprites dataset contains three shapes (a square, an ellipse and a heart) with varying *x* and *y* positions, orientations and scale information. In addition to these two synthetic datasets, we also use three real-world datasets from three different scientific domains, namely, EMPIAR-10029^22^ (from life sciences), graphene CBED pattern^2^ (from material science) and Galaxy-Zoo^17^ (from astronomy) datasets.

### CODAE model

With the enhanced encoder design described in ‘CODAE architecture’ section, CODAE consists of five components: a translation- and rotation-equivariant encoder, Euler encoding, a spatial transformation, a standard decoder, denoted by *D*_*θ*_, that uses conventional 2D convolutional layers, and an image moment loss. The translation- and rotation-equivariant encoder takes images, *I*, as inputs. Next, the features derived from the equivariant layers are passed to two branches. The first branch *B*_equi_ outputs (*x*, *y*)-position and orientation features, which we denote by **z**_equi_. The second branch, *B*_inv_, outputs invariant features, which we denote by **z**_inv_. Subsequently, we use Euler encoding^7^, denoted by *E*, which enforces orthogonality in the latent space, enabling each dimension in the latent space to be uncorrelated. Therefore, applying Euler encoding to **z**_inv_ enables learning disentangled, translation- and rotation-invariant features. These features are then fed into the decoder to output images aligned with each other. Finally, the aligned image is generated from *D*_*θ*_ and translated and rotated using a spatial transformation using **z**_equi_. To learn centroids and orientations while reconstructing the input images, CODAE minimizes the following losses:

1 $${L}_{{\mathrm{r}}}(I,{T}_{{{\boldsymbol{z}}}_{{\mathrm{equi}}}}({D}_{{{\theta }}}(E({{\mathbf{z}}}_{{\mathrm{inv}}}))))+\beta {L}_{{\mathrm{m}}}(\{\bar{x},\bar{y},\bar{\alpha }\},{{\mathbf{z}}}_{{\mathrm{equi}}}),$$

where ${T}_{{{\mathbf{z}}}_{{\mathrm{equi}}}}$ is a spatial transformation using **z**_equi_. While the *L*_r_ loss reduces the difference between the input and reconstructed images, the *L*_m_ loss minimizes the difference between the image moments and **z**_equi_. The value of *β* gradually decreases from large to zero (Supplementary Equation 9), guiding the model to learn the centroid and orientation extracted using image moments during its initial learning stage.

### Baseline models

We compare CODAE with several baselines that either disentangle features or explicitly learn centroids and orientations of images, enabling the reconstruction of aligned images. *β*-VAE^6,34^ and DAE^7^ are designed to disentangle features. While the former introduces statistical independence to the network by assuming the prior distribution of the latent space to be Gaussian, the latter incorporates an orthogonal transformation in the latent space, enabling output changes from changes of different latent dimensions to be orthogonal. Spatial-VAE^1^, target-VAE^10^ and IRL-INR^11^ are proposed to learn centroids and orientations of images explicitly. Spatial-VAE, based on VAE, utilizes a spatial decoder that maps spatial coordinates to pixel values^1^. The spatial decoder takes a grid and *N* features from the model’s encoder in this framework. It uses three dominant features to translate and rotate the spatial coordinates in the grid. Subsequently, the pixel values are decided on the basis of the coordinates and the remaining *N* − 3 features. However, while spatial-VAE can partially reconstruct aligned images for certain datasets, such as proteins and galaxies, the approach fails on some, such as the Modified National Institute of Standards and Technology (MNIST) dataset, owing to the symmetry issues when digits are rotated through angles ranging between −180° and 180°. The translation and rotation group-equivariant VAE framework (target-VAE) addresses this issue by designing an improved encoder that outputs attention values, angles and content components^10^. This encoder consists of group convolution layers proposed in refs. ^23,33^. The attention values assist in learning rotation angles from the discretized rotation component, effectively resolving the issue in spatial-VAE. The IRL-INR approach also maps the spatial coordinates to pixel values, with the model containing an encoder and a hypernetwork that parameterizes images^11^. Learning centroids and orientations separately use two additional loss functions that force the model to learn invariant features.

### Metrics

Numerous metrics have been proposed to quantitatively assess the degree of disentanglement. In our study, we adopt the notions introduced in ref. ^24^, where the metrics are divided into three classes based on their fundamental approaches, namely, intervention-based, predictor-based, and information-based metrics. For intervention-based metrics, we use z-diff^6^, z-var^25^ and irs^26^. z-diff quantifies the difference in each latent variable about a target factor. A smaller difference indicates that the latent variables consistently represent the target factor across different inputs. z-var measures the variance of each latent dimension when a target factor is fixed. Lower variance reflects that each dimension aligns with a specific factor. irs (interventional robustness score) evaluates the stability of latent dimensions assigned to specific factors by calculating distances between encoded representations before and after modifying non-target factors. A lower IRS indicates that variations in unrelated attributes within the data do not affect the model. In the category of predictor-based metrics, we include dci^27^ and sap^28^. dci (disentanglement, compactness and informativeness) uses a lasso regressor or random forest to generate a feature importance matrix, reflecting the relationship between each latent variable and specific factors. sap (separated attribute predictability) measures scores on the basis of the prediction of each factor from individual latent dimensions. A larger difference between the two highest scores suggests that a single latent dimension effectively encodes a specific factor. Lastly, for information-based metrics, we use mig^29^, jemmig^30^ and dcimig^31^. mig, or mutual information gap, measures the difference between the highest and second-highest mutual information values for a fixed factor, ensuring that each factor is primarily encoded in a single latent dimension. jemmig, or joint entropy minus mutual information gap, extends mig by incorporating joint entropy, addressing the limitation of mig, where two factors may be encoded in a single latent dimension. Similarly, dcimig combines insights from dci and mig to address the limitation in mig. It calculates mig between factors and latent dimensions, then assigns each factor a score based on all associated migs, reflecting the independence of each factor representation within the latent space. These metrics are all applied in a supervised manner, normalized between 0 and 1 (with 1 indicating optimal performance) and capture three main properties of disentangled representations: modularity, compactness and explicitness, for the XYRCS and dSprites datasets.

## Supplementary information

Supplementary Information Supplementary Figs. 1–6, details of methods and experimental results, and Tables 1–8.

## Data Availability

We used the XYRCS and dSprites datasets that contain reliable ground-truth labels^7,19^. The XYRCS dataset is a simple yet effective synthetic dataset containing three shapes (a circle, a triangle and a rectangle) with varying *x* and *y* positions, orientations and colour information (specifically, the brightness). The dSprites dataset contains three shapes (a square, an ellipse and a heart) with varying *x* and *y* positions, orientations and scale information. In addition to these two synthetic datasets, we also use three real-world datasets from three different scientific domains, namely, EMPIAR-10029^22^ (from life sciences), graphene CBED pattern^2^ (from material science) and Galaxy-Zoo^17^ (from astronomy) datasets.

## Code availability

The source code for this study, along with the relevant documentation, is available via Code Ocean at 10.24433/CO.2120714.v1 with the MIT licence^32–34^.
