# Supplementary material for: Discovering fully semantic representations via centroid- and orientation-aware feature learning
Source: Nat Mach Intell. 2025 Feb 6;7(2):307–14. doi: 10.1038/s42256-024-00978-5 (PMC11847704; doi:10.1038/s42256-024-00978-5)
Supplement: Supplementary file 1 — Supplementary Figs. 1–6, details of methods and experimental results, and Tables 1–8. [file 42256_2024_978_MOESM1_ESM.pdf]

nature machine intelligence

Supplementary information

https://doi.org/10.1038/s42256-024-00978-5

Discovering fully semantic representations via centroid- and orientation-aware feature learning

In the format provided by the authors and unedited

A Supplementary

A.1 Theoretical foundations for establishing both translational and rotational equivariant and invariant layers

It is well known that the conventional two-dimensional convolutional layer inherently possesses translational equivariance. We introduce supplementary operations to the conventional convolution process to obtain equivariance in translation and rotation. In the sequel, we consider functions $f \in L^p(\mathbb{R}^2)$ and $g \in L^q(\mathbb{R}^2)$, $\frac{1}{p} + \frac{1}{q} = 1 (p \geq 1)$, so that $\int_{\mathbb{R}^2} f(\boldsymbol{x}) g(\boldsymbol{x})\, d\boldsymbol{x} < \infty$.

Theorem 1. *For functions $f \in L^p(\mathbb{R}^2)$ and $g \in L^q(\mathbb{R}^2)$, let $L_{\boldsymbol{t}}$ be a function defined by $(L_{\boldsymbol{t}} f)(\boldsymbol{x}) = f(\boldsymbol{x} - \boldsymbol{t})$, for $\boldsymbol{x}, \boldsymbol{t} \in \mathbb{R}^2$. Then the 2D-convolution of $L_{\boldsymbol{t}} f$ and g, denoting the operator by the symbol $*$, is translational equivariant [33].*

Theorem 1 shows that the conventional two-dimensional convolution satisfies the translational equivariance property. We now introduce supplementary operations using the convolution above to obtain rotational equivariance. In order to have an operation that is rotational equivariance, we consider a function $\tilde{L}_\phi$ exhibiting rotation by $(\tilde{L}_\phi f)(\boldsymbol{x}) = f(R_{-\phi}\boldsymbol{x})$ for $\boldsymbol{x} \in \mathbb{R}^2$ and $\phi \in \mathbb{R}$, where

$$R_\phi = \begin{bmatrix} \cos(\phi) & -\sin(\phi) \\ \sin(\phi) & \cos(\phi) \end{bmatrix}. \tag{2}$$

Theorem 2. *Let $\tilde{*}$ be an operation defined by $(f \tilde{*} g)(\boldsymbol{x}) = \int_{S^1} \int_{\mathbb{R}^2} f(\boldsymbol{y}) g(R_{-\theta}(\boldsymbol{x} - \boldsymbol{y}))\, d\boldsymbol{y}\, d\theta$. Then $\tilde{*}$ is equivariant under both translations and rotations.*

Proof(a) From Theorem 1, it is easy to see that $\tilde{*}$ is translational equivariant.
(b) For $\boldsymbol{x} \in \mathbb{R}^2$ and $\psi \in \mathbb{R}$, we obtain that

$$\begin{aligned}
[(\tilde{L}_\phi f) \tilde{*} g](\boldsymbol{x}) &= \int_{S^1} \int_{\mathbb{R}^2} (L_\phi f)(\boldsymbol{y}) g(R_{-\theta}(\boldsymbol{x} - \boldsymbol{y}))\, d\boldsymbol{y}\, d\theta \\
&= \int_{S^1} \int_{\mathbb{R}^2} f(R_{-\phi}\boldsymbol{y}) g(R_{-\theta}(\boldsymbol{x} - \boldsymbol{y}))\, d\boldsymbol{y}\, d\theta \\
&= \int_{S^1} \int_{\mathbb{R}^2} f(\tilde{\boldsymbol{y}}) g(R_{-\theta}(\boldsymbol{x} - R_\phi \tilde{\boldsymbol{y}}))\, d\tilde{\boldsymbol{y}}\, d\theta \\
&= \int_{S^1} \int_{\mathbb{R}^2} f(\tilde{\boldsymbol{y}}) g(R_{-\theta} R_\phi (R_{-\phi}\boldsymbol{x} - \tilde{\boldsymbol{y}}))\, d\tilde{\boldsymbol{y}}\, d\theta \\
&= \int_{S^1} \int_{\mathbb{R}^2} f(\tilde{\boldsymbol{y}}) g(R_{-\tilde{\theta}} (R_{-\phi}\boldsymbol{x} - \tilde{\boldsymbol{y}}))\, d\tilde{\boldsymbol{y}}\, d\tilde{\theta} \\
&= (f \tilde{*} g)(R_{-\phi}\boldsymbol{x}) = \tilde{L}_\phi (f \tilde{*} g)(\boldsymbol{x}),
\end{aligned} \tag{3}$$

where $\tilde{\boldsymbol{y}} = R_{-\phi}(\boldsymbol{y})$ and $\tilde{\theta} = \theta - \phi$.

□

Theorem 3. *Let f be a function on $\mathbb{R}^2$ such that $\sup_{\boldsymbol{x} \in \mathbb{R}^2} f(\boldsymbol{x}) < \infty$. Then the supremum of the function is invariant under both translations and rotations.*

Proof. Let $M = \sup_{\boldsymbol{x}\in\mathbb{R}^2} f(\boldsymbol{x})$. Then for any $\boldsymbol{t} \in \mathbb{R}^2$ and $\phi \in \mathbb{R}$, it is easy to check that $M = \sup_{\boldsymbol{x}\in\mathbb{R}^2} f(\boldsymbol{x} - \boldsymbol{t}) = \sup_{\boldsymbol{x}\in\mathbb{R}^2} f(R_\phi \boldsymbol{x})$. Hence M is invariant under translations and rotations. □

We note that $\sup_{\boldsymbol{x}\in\mathbb{R}^2} f(\boldsymbol{x}) = \max_{\boldsymbol{x}\in\mathbb{R}^2} f(\boldsymbol{x})$ if f is a continuous function which has a compact support, i.e. the closure of $\{\boldsymbol{x} \in \mathbb{R}^2 : f(\boldsymbol{x}) \neq 0\}$ is closed and bounded.

The translational and rotational equivariant layers are implemented based on [23], primarily for the reasons of seeking an efficient implementation.

A.2 Image Moments

Here, we present the relevant background regarding image moments. Let $f(x, y)$ be a 2D-continuous function. Then the moment of order $(p + q)$ is defined as

$$M_{pq} = \int_{-\infty}^{\infty}\int_{-\infty}^{\infty} x^p y^q f(x, y)\, dx\, dy \tag{4}$$

for $p, q \in \mathbb{Z}^+ \cup \{0\}$. For a gray-scale image I, M_{00} represents an area of I, and the centroid of I can be represented by the first order moments as $(\bar{x}, \bar{y}) = (\frac{M_{10}}{M_{00}}, \frac{M_{01}}{M_{00}})$. Furthermore, in order to describe the shape of the function without considering translation, we can use central moments defined as

$$\mu_{pq} = \int_{-\infty}^{\infty}\int_{-\infty}^{\infty} (x - \bar{x})^p (y - \bar{y})^q f(x, y)\, dx\, dy. \tag{5}$$

Then the second order central moments are used to extract the orientation of I. First, we calculate the covariance matrix of I,

$$Cov(I) = \begin{bmatrix} c_{20} & c_{11} \\ c_{11} & c_{02} \end{bmatrix}. \tag{6}$$

where $c_{ij} = \frac{\mu_{ij}}{\mu_{00}}$. Since the eigenvectors of this matrix correspond to the directions of maximum and minimum variance of image intensity, the orientation can be extracted from the angle formed between the eigenvector associated with the largest eigenvalue and the axis that is closest to this eigenvector. Therefore, the orientation α can be expressed by

$$\alpha = \frac{1}{2} \arctan\left(\frac{2c_{11}}{c_{20} - c_{02}}\right). \tag{7}$$

However, this orientation only represents the specific angle formed between the eigenvector associated with the largest eigenvalue and the nearest axis. Therefore, two images flipped by any line that passes through the center will have the same orientation value as discussed in Prokop and Reeves [13]. To address this issue, Prokop and Reeves [13] further used the third-order moments to capture the skewness of the image. The skewness represents how the pixel intensities are distributed asymmetrically around the mean intensity. This value distinguishes between two mirror images exhibiting symmetry and possessing opposite skewness signs. In this study, we first align the image, I, with the eigenvector using the value α in Eq. 7 to obtain an aligned

image, denoted by $\tilde{I}$. Then, one of the third order moments of $\tilde{I}$, which we denote $\tilde{M}_{30}$, is calculated to describe the skewness of $\tilde{I}$ along the x-axis. Since the rotation of an image by 180° changes the sign of the skewness of the projection on the x-axis, it can distinguish the two mirror images. Therefore, we assign the orientation of I by

$$\bar{\alpha} = \begin{cases} \alpha, & \text{if } \tilde{M}_{30} > 0 \\ \alpha + \pi, & \text{otherwise.} \end{cases} \tag{8}$$

Table S1 Comparison between CODAE and Image Moments for disentanglement scores in centroid and orientation learning on the XYRCS and dSprites datasets. In this analysis, we use only the labels and features corresponding to x position, y position, and rotation angle to calculate disentanglement scores. A higher value is preferred across all metrics.

Datasets	Models	z-diff	z-var	irs	dci	sap	mig	jemmig	dcimig	avg
XYRCS	CODAE	1.00	0.83	0.64	0.65	0.44	0.59	0.73	0.59	0.68
	Image moments	1.00	0.74	0.23	0.71	0.51	0.50	0.68	0.52	0.61
dSprties	CODAE	1.00	1.00	0.83	0.30	0.66	0.45	0.63	0.43	0.66
	Image moments	1.00	0.86	0.43	0.35	0.52	0.42	0.62	0.40	0.57

As we mentioned in Section 1.1, image moments can exhibit subtle inaccuracies due to several factors. First, they are sensitive to noise because the moments are calculated from pixel intensities, making them susceptible to small variations that can lead to inaccuracies. Additionally, image moments capture global shape characteristics across entire objects, which can result in a loss of local features, especially when finer rotational details are essential. Table S1 demonstrates that CODAE enhances centroid and orientation learning. For Image Moments, the disentanglement score is calculated by replacing representations generated by CODAE with approximated centroids and orientations derived from image moments.

A.3 Reconstructions of latent traversals across each latent dimension

XYRCS dataset. The reconstructions of latent traversal across each latent dimension obtained by all models are shown in Figure S1. While all models can disentangle x position, y position, and color from the other features, DAE and β-VAE do not exhibit rotation using one feature. In this experiment, Spatial-VAE disentangles all five factors while Target-VAE fails to produce rectangles, making it difficult to check whether the model successfully learns all factors. IRL-INR has another difficulty in separating the color and shape factors.

dSprites dataset. Similar to the XYRCS dataset, all models can disentangle the x position and y position. However, CODAE and Spatial-VAE only separate the scale and shape factors. Although the disentanglement scores of β-VAE are relatively high in Table S3, its reconstruction performance is unsatisfactory, as shown in Figure 4 and

Figure S2, which is widely acknowledged by Burgess et al. [34]. Most models obtain lower disentanglement scores in the dSprties dataset than the XYRCS dataset, and the latent traversals also show the inabilities of each model to disentangle the dSprites dataset. Spatial-VAE and IRL-INR exhibit the ellipse rotation and shape variation, while Target-VAE cannot reconstruct the clear images.

EMPIAR-10029 dataset. As CODAE, Spatial-VAE, and Target-VAE can align the input data, they learn semantic representations such as background and out-of-plane orientations as shown in Figure S3. The failure of the alignment of the input images by IRL-INR shows that the model fails to fit all the semantic representations.

Graphene CBED pattern dataset. While all models learn rotation features, Spatial-VAE and Target-VAE fail to reconstruct clear and sharp images, as shown in Figure S4. We use a three-dimensional latent space in this dataset. Given the importance of diffraction patterns in analyzing Graphene CBED patterns, we also show the diffraction patterns generated from the learned representations of each model in Figure S6. To derive these patterns, we first calculate the Pearson correlation coefficient between the latent features and the variables COMx, COMy, COMangle, and COMmag. For each variable, we select the latent feature with the highest correlation score. Once the representations corresponding to COM_x, COM_y, COM_{angle}, and COM_{mag} are identified, we use these features to plot the diffraction patterns.

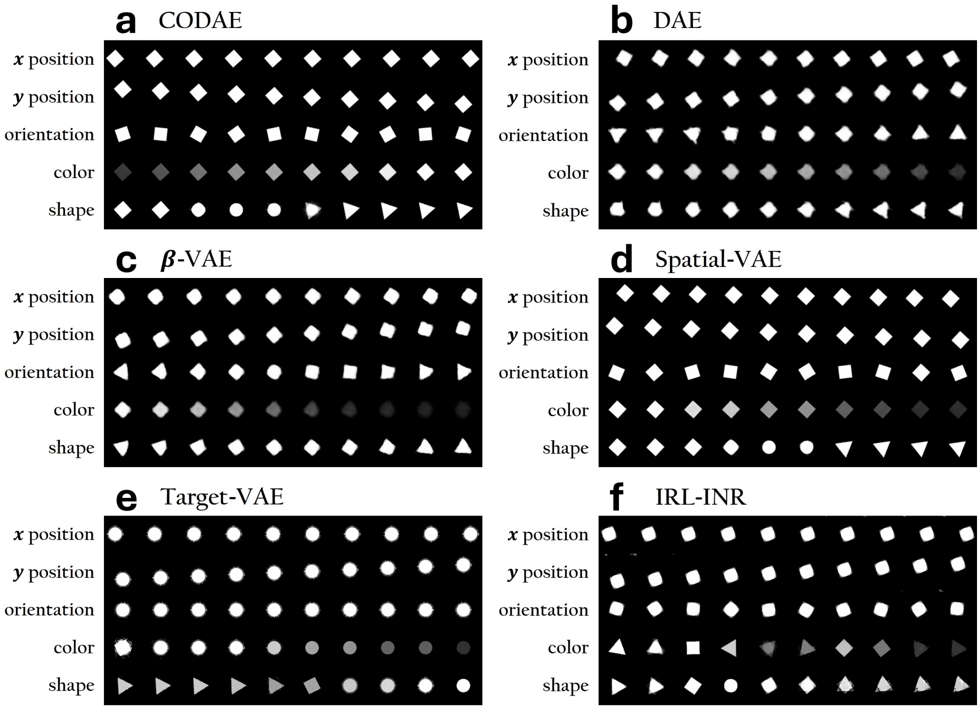

Fig. S1 Reconstructions of latent traversal across each latent dimension obtained by CODAE, DAE, β-VAE, Spatial-VAE, Target-VAE, and IRL-INR for the XYRCS dataset.

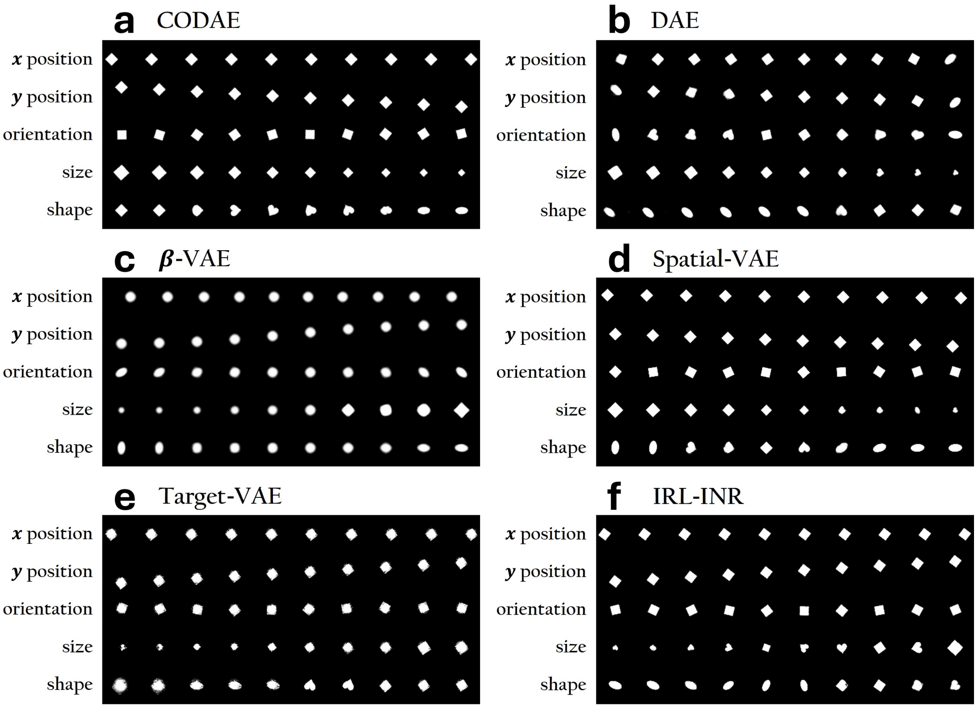

Fig. S2 Reconstructions of latent traversal across each latent dimension obtained by CODAE, DAE, β-VAE, Spatial-VAE, Target-VAE, and IRL-INR for the dSprites dataset.

Galaxy-Zoo dataset. CODAE is the only model that aligns input images with high quality, as shown in Figure 4; as a result, other models fail to learn some of the semantic representations. Significantly, no models learn separation other than CODAE, as shown in Figure S5. While Spatial-VAE learns some aspects of the images, Target-VAE and IRL-INR only learn the x position and y position.

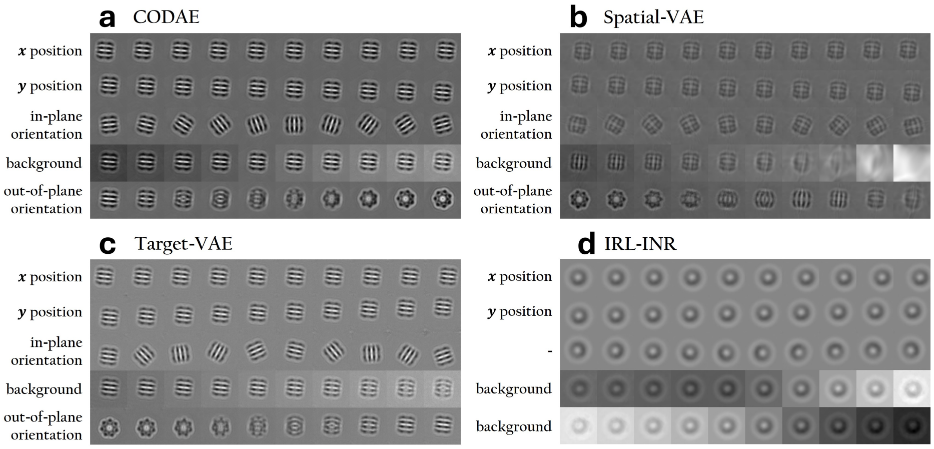

Fig. S3 Reconstructions of latent traversal across each latent dimension obtained by CODAE, DAE, β-VAE, Spatial-VAE, Target-VAE, and IRL-INR for the EMPIAR-10029 dataset.

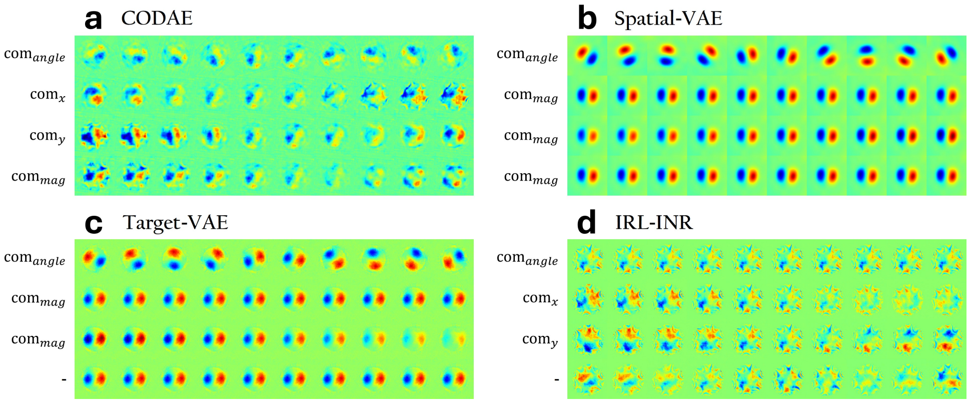

Fig. S4 Reconstructions of latent traversal across each latent dimension obtained by CODAE, DAE, β-VAE, Spatial-VAE, Target-VAE, and IRL-INR for the Graphene CBED pattern dataset.

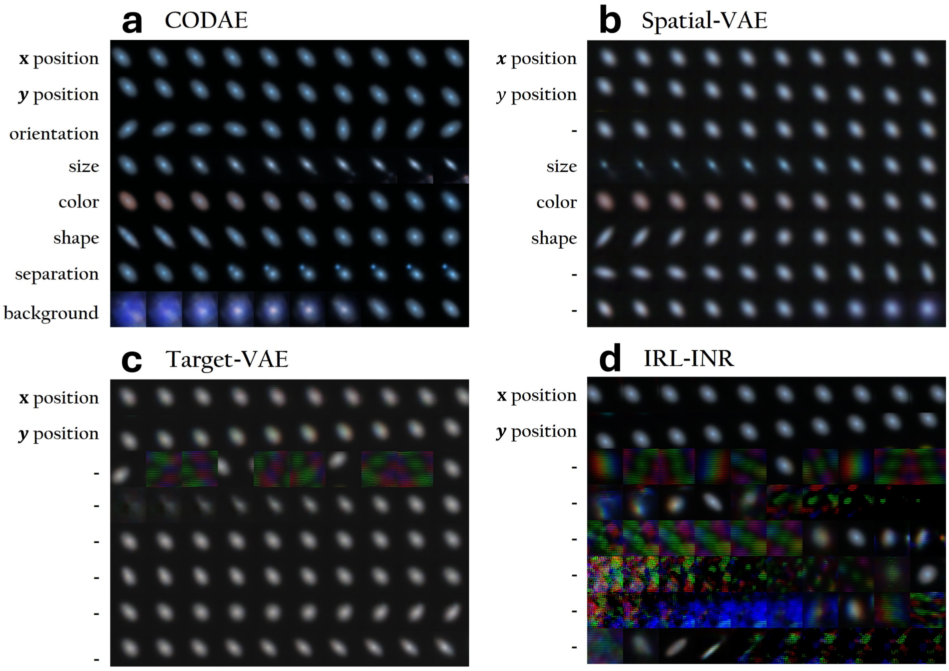

Fig. S5 Reconstructions of latent traversal across each latent dimension obtained by CODAE, DAE, β-VAE, Spatial-VAE, Target-VAE, and IRL-INR for the Galaxy-Zoo dataset.

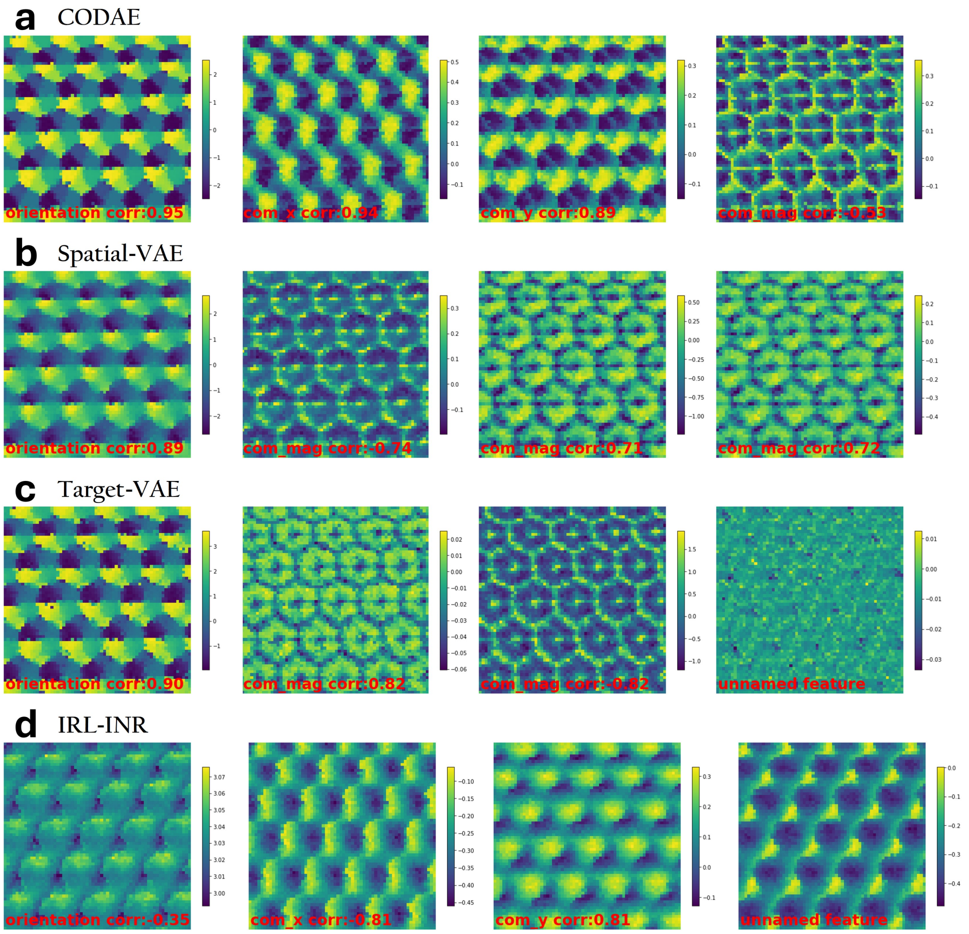

Fig. S6 COM_x, COM_y, COM_{angle} and COM_{mag} of diffraction patterns from each feature with the Pearson correlation coefficient by CODAE, Spatial-VAE, Target-VAE, and IRL-INR for the Graphene CBED pattern dataset. We use the Pearson correlation coefficient of the latent features with COM_x, COM_y, COM_{angle} and COM_{mag} and select the feature with the highest score for each feature.

A.4 Table of disentanglement scores and ablation study.

In the table below, for the features in XYRCS, X, Y, R, C, and S represent the x position, y position, rotation angle, color, and shape, respectively. For the dSprites dataset, X, Y, R, S, and S represent the x position, y position, rotation angle, scale, and shape, respectively. For ablation study, we compare CODAE with DAE+translational and rotational equivariant encoder (TREE) and DAE+L_{moment}. CODAE can be viewed as DAE+TREE+L_{moment}. This comparison allows us to assess, for example, the performance drop when comparing CODAE with DAE+TREE, which shows the impact of removing L_{moment} and using only L_{recon}.

Table S2 Disentanglement scores for the XYRCS dataset. A higher value is preferred across all metrics.

Features	Models	z-diff	z-var	irs	dci	sap	mig	jemmig	dcimig	avg
XYRCS	CODAE	**0.97**	0.76	0.67	**0.83**	0.61	**0.67**	**0.72**	**0.63**	**0.73**
	DAE	0.89	0.80	**0.87**	0.66	0.63	0.48	0.64	0.53	0.68
	β-VAE	0.74	0.71	0.64	0.32	0.51	0.21	0.45	0.25	0.47
	Spatial-VAE	0.79	0.60	0.54	0.55	0.47	0.44	0.50	0.28	0.52
	Target-VAE	0.95	**0.86**	0.58	0.62	**0.74**	0.47	0.65	0.50	0.67
	IRL-INR	0.79	0.61	0.59	0.41	0.38	0.31	0.47	0.28	0.48
CS	CODAE	**1.00**	**1.00**	**0.82**	**0.99**	**0.83**	**0.90**	0.77	**0.90**	**0.90**
	DAE	**1.00**	**1.00**	0.68	0.77	0.48	0.64	0.64	0.72	0.74
	β-VAE	0.61	**1.00**	0.52	0.31	0.34	0.28	0.43	0.28	0.47
	Spatial-VAE	**1.00**	**1.00**	0.74	0.96	0.80	0.84	**0.78**	0.88	0.87
	Target-VAE	**1.00**	**1.00**	0.60	0.65	0.81	0.55	0.60	0.54	0.71
	IRL-INR	0.87	0.51	0.56	0.72	0.26	0.52	0.53	0.52	0.56

Table S3 Disentanglement scores for the dSprties dataset. A higher value is preferred across all metrics.

Features	Models	z-diff	z-var	irs	dci	sap	mig	jemmig	dcimig	avg
XYRSS	CODAE	**0.97**	**0.85**	**0.82**	**0.56**	**0.59**	**0.48**	**0.64**	**0.45**	**0.67**
	DAE	0.86	0.64	0.63	0.37	0.56	0.33	0.49	0.28	0.52
	β-VAE	0.80	0.69	**0.82**	0.50	0.57	0.36	0.57	0.36	0.58
	Spatial-VAE	0.67	0.48	0.57	0.32	0.31	0.20	0.37	0.15	0.38
	Target-VAE	0.88	0.78	0.54	0.36	0.54	0.37	0.51	0.32	0.53
	IRL-INR	0.89	0.72	0.69	0.45	0.45	0.28	0.51	0.25	0.53
SS	CODAE	**1.00**	0.99	**0.74**	**0.89**	**0.51**	**0.58**	**0.71**	**0.64**	**0.75**
	DAE	0.93	**1.00**	0.49	0.53	0.46	0.45	0.54	0.48	0.61
	β-VAE	0.94	0.46	0.47	0.51	0.49	0.53	0.57	0.57	0.56
	Spatial-VAE	0.92	**1.00**	0.56	0.53	0.50	0.42	0.55	0.45	0.61
	Target-VAE	0.90	0.82	0.54	0.46	0.41	0.48	0.55	0.42	0.57
	IRL-INR	0.99	0.99	0.65	0.45	0.44	0.35	0.59	0.40	0.60

Table S4 Ablation study of CODAE. Disentanglement scores for the XYRCS dataset with only L_{moment} or the translational and rotational equivariant encoder (TREE).

Features	Models	z-diff	z-var	irs	dci	sap	mig	jemmig	dcimig	avg
XYRSS	CODAE	0.97	0.76	0.67	0.83	0.61	0.67	0.72	0.63	0.73
	DAE+TREE	0.82	0.73	0.59	0.48	0.37	0.22	0.48	0.24	0.49
	DAE+L_{moment}	0.86	0.74	0.87	0.57	0.63	0.44	0.61	0.51	0.65
SS	CODAE	1.00	1.00	0.82	0.99	0.83	0.90	0.77	0.90	0.90
	DAE+TREE	0.92	0.81	0.38	0.35	0.13	0.16	0.47	0.16	0.42
	DAE+L_{moment}	0.57	0.78	0.45	0.20	0.00	0.01	0.29	0.01	0.28

Table S5 Ablation study of CODAE. Disentanglement scores for the dSprites dataset with only L_{moment} or the translational and rotational equivariant encoder (TREE).

Features	Models	z-diff	z-var	irs	dci	sap	mig	jemmig	dcimig	avg
XYRCS	CODAE	0.97	0.85	0.82	0.56	0.59	0.48	0.64	0.45	0.67
	DAE+TREE	0.92	0.78	0.65	0.36	0.34	0.30	0.45	0.21	0.50
	DAE+L_{moment}	0.71	0.56	0.42	0.24	0.33	0.19	0.37	0.14	0.37
CS	CODAE	1.00	0.99	0.74	0.89	0.51	0.58	0.71	0.64	0.75
	DAE+TREE	0.95	0.83	0.29	0.61	0.38	0.55	0.61	0.53	0.59
	DAE+L_{moment}	0.84	0.53	0.34	0.33	0.44	0.40	0.46	0.38	0.46

A.5 Training Details

To achieve the best numerical scores and reconstructions, all models are trained with four different learning rates: 0.001, 0.0005, 0.0001, and 0.00005 across all datasets. Each model is run for 100 epochs across the XYRCS, dSprites, and EMPIAR-10029 datasets. For the Galaxy-Zoo dataset, Spatial-VAE and Target-VAE are trained for 200 epochs, and the IRL-INR is trained for 300 epochs to guarantee that their reconstruction losses are small enough for comparison. For the Graphene CBED pattern dataset, all models are performed for 200 epochs for the same reason as the Galaxy-Zoo dataset.

In all experiments, for β in the loss function of CODAE, we use four values: 1, 10, 50, and 100. The optimal β varies across different datasets as shown in Table S6. β gradually decreases and reaches zero after a pre-defined final epoch, according to the following schedule:

$$\beta_i = \beta_{initial} \cdot (1 - \frac{\gamma}{N})^i \tag{9}$$

where $\beta_{initial}$ is the initial β, γ is decay rate and N is the number of iteration in a single epoch. The final epochs for β across the different datasets also can be found in Table S6.

Table S6 The value β used in CODAE for different datasets.

Dataset	β	Final epoch
XYRCS	1	50
dSprites	1	10
EMPIAR-10029	1	50
Galaxy-Zoo	10	1
CBED pattern	50	100

A.6 Training and Inference speed on different GPU models

The proposed model uses the standard convolutional layers, while the other centroid and orientation learning models use fully connected layers or hypernetworks in their decoder parts. Therefore, the proposed model maintains the computational cost to the minimum. We verify this by measuring the training and inference time on three different GPU architectures, namely, $V100, A100$, and $H100$. We provide an average of ten runs. Table S7 shows that the other models' training and inference speeds are rapidly decreasing along with the dimension of images. Additionally, we report the number of parameters for each model across different datasets. To ensure a fair comparison, we used similar model hyperparameters for Spatial-VAE, Target-VAE, and IRL-INRas specified in [1, 10, 11], thereby avoiding any bias toward CODAE. Table S8 indicates that CODAE consistently ranks among the two smallest models across all datasets.

Table S7 Training and inference iterations per second for each model across different datasets on different GPU architectures. A higher value is preferred.

		V100		A100		H100	
Datasets	Models	training	inference	training	inference	training	inference
XYRCS ($84 \times 84 \times 1$) Batch size: 64	CODAE	**43.24**	**98.55**	**45.67**	**99.61**	**66.53**	**134.01**
	Spatial-VAE	11.50	28.75	35.36	74.62	27.50	71.92
	Target-VAE	2.02	4.21	4.21	8.07	4.29	11.61
	IRL-INR	1.16	6.66	2.08	11.84	2.84	17.78
dSprites CBED pattern ($64 \times 64 \times 1$) Batch size: 64	CODAE	**37.26**	**86.75**	41.76	99.27	**63.67**	**135.35**
	Spatial-VAE	19.64	48.00	**58.45**	**116.13**	48.32	128.84
	Target-VAE	3.73	10.12	8.65	19.36	9.88	26.55
	IRL-INR	1.67	9.32	2.78	13.51	3.82	21.29
Galaxy-Zoo ($64 \times 64 \times 3$) Batch size: 64	CODAE	**22.01**	**55.42**	**29.56**	**61.68**	**48.35**	**99.62**
	Spatial-VAE	1.08	2.47	1.74	4.25	1.82	4.57
	Target-VAE	3.67	8.59	7.54	14.26	6.14	10.48
	IRL-INR	1.67	9.23	2.76	13.05	6.79	20.42
EMPIAR-10029 ($128 \times 128 \times 1$) Batch size: 32	CODAE	**20.43**	**58.46**	**25.79**	**57.78**	**40.19**	**124.82**
	Spatial-VAE	1.76	1.25	1.06	2.21	1.05	2.27
	Target-VAE	1.21	2.27	3.02	4.08	3.00	7.56
	IRL-INR	1.68	7.55	3.23	14.29	4.24	20.12

Table S8 Number of parameters for each model across different datasets

Datasets	Models	The number of parameters
XYRCS	CODAE	128078
	Spatial-VAE	4146699
	Target-VAE	1035400
	IRL-INR	79724998
dSprites	CODAE	383382
	Spatial-VAE	2631179
	Target-VAE	940680
	IRL-INR	79724998
CBED pattern	CODAE	383511
	Spatial-VAE	575881
	Target-VAE	941450
	IRL-INR	79725767
Galaxy-Zoo	CODAE	5102143
	Spatial-VAE	6845459
	Target-VAE	2385808
	IRL-INR	79861069
EMPIAR-10029	CODAE	5903526
	Spatial-VAE	8929291
	Target-VAE	1333896
	IRL-INR	79724998
